# Combining Mobile Crowdsensing and Ecological Momentary Assessments in the Healthcare Domain

**DOI:** 10.3389/fnins.2020.00164

**Published:** 2020-02-28

**Authors:** Robin Kraft, Winfried Schlee, Michael Stach, Manfred Reichert, Berthold Langguth, Harald Baumeister, Thomas Probst, Ronny Hannemann, Rüdiger Pryss

**Affiliations:** ^1^Institute of Databases and Information Systems, Ulm University, Ulm, Germany; ^2^Department of Clinical Psychology and Psychotherapy, Ulm University, Ulm, Germany; ^3^Clinic and Policlinic for Psychiatry and Psychotherapy, University of Regensburg, Regensburg, Germany; ^4^Department for Psychotherapy and Biopsychosocial Health, Danube University Krems, Krems an der Donau, Austria; ^5^Sivantos GmbH, Erlangen, Germany; ^6^Institute of Clinical Epidemiology and Biometry, University of Würzburg, Würzburg, Germany

**Keywords:** mobile crowdsensing (MCS), crowdsourcing, ecological momentary assessments (EMA), mobile healthcare application, chronic disorders, reference architecture

## Abstract

The increasing prevalence of smart mobile devices (e.g., smartphones) enables the combined use of mobile crowdsensing (MCS) and ecological momentary assessments (EMA) in the healthcare domain. By correlating qualitative longitudinal and ecologically valid EMA assessment data sets with sensor measurements in mobile apps, new valuable insights about patients (e.g., humans who suffer from chronic diseases) can be gained. However, there are numerous conceptual, architectural and technical, as well as legal challenges when implementing a respective software solution. Therefore, the work at hand (1) identifies these challenges, (2) derives respective recommendations, and (3) proposes a reference architecture for a MCS-EMA-platform addressing the defined recommendations. The required insights to propose the reference architecture were gained in several large-scale mHealth crowdsensing studies running for many years and different healthcare questions. To mention only two examples, we are running crowdsensing studies on questions for the tinnitus chronic disorder or psychological stress. We consider the proposed reference architecture and the identified challenges and recommendations as a contribution in two respects. First, they enable other researchers to align our practical studies with a baseline setting that can satisfy the variously revealed insights. Second, they are a proper basis to better compare data that was gathered using MCS and EMA. In addition, the combined use of MCS and EMA increasingly requires suitable architectures and associated digital solutions for the healthcare domain.

## 1. Introduction

For many use cases in the healthcare domain, e.g., in the assessment of chronic diseases and disorders, there is a need for the collection of large, qualitative, longitudinal, and ecologically valid data sets. Additionally, contextual information like environmental factors can give even more valuable insights to researchers, healthcare providers (e.g., physicians or therapists), and last but not least, the patients themselves. At the same time, smart mobile devices (e.g., smartphones and smartwatches) and low-powered sensors are becoming increasingly ubiquitous. Two concepts that highly benefit from these advancements are *mobile crowdsensing* (MCS) and *ecological momentary assessments* (EMA). They can be used in combination in the form of mobile apps to correlate EMA assessment data with sensor measurement data in order to gain even more valuable insights about patients. However, there are numerous challenges when implementing a software solution in order to provide the desired functionality, to cope with technical aspects, as well as to comply with high standards and regulations in the healthcare domain. In this work, we discuss these challenges, derive several recommendations and propose a reference architecture for a respective software platform. These insights were mainly gained through several studies that combined MCS and EMA based on mHealth apps that we have developed in the last years. The mentioned studies, in turn, address different healthcare questions and are mostly running for many years. This provides us with a proper basis for the proposed reference architecture as well as the introduced set of recommendations. To conclude, the work at hand provides the following contributions:

- Various challenges are pointed out and discussed on the basis of the ongoing research project TrackYourTinnitus (TYT), which has been running since 2014.
- A number of recommendations are derived from the findings during this and other related projects.
- A reference architecture for a platform enabling the combination of MCS and EMA is proposed that aims to address the defined recommendations. Additionally, technical considerations for the implementation of the architecture are discussed.

The remainder of this paper is organized as follows. In section 2, related work in the fields of mobile crowdsensing and ecological momentary assessments is presented, and the combination of both concepts is discussed. Lessons learned during the operation of the TrackYourTinnitus (TYT) project are presented in section 3. In section 4, we derive recommendations for a MCS-EMA platform, propose a reference architecture to address these recommendations, and discuss selected technical considerations. Furthermore, the findings and their implications for MCS and EMA research are discussed in section 5. Finally, section 6 concludes the paper with a summary and an outlook.

## 2. Mobile Crowdsensing in Healthcare

In this section, we discuss mobile crowdsensing (MCS) in the healthcare domain. We cover related work in the fields of MCS and EMA and explain how we relate ecological momentary assessments (EMA) apps to MCS.

### 2.1. Mobile Crowdsensing (MCS)

*Mobile crowdsensing* is a paradigm in which a community is leveraging devices with sensing and computing capabilities to collectively share data and extract information in order to measure and map phenomena of common interest. Therefore, it is also referred to as *community sensing*. As opposed to *personal sensing*, where the phenomena that are monitored belong to an individual user, community sensing applications focus on monitoring large-scale phenomena that cannot easily be measured by a single user or device (Ganti et al., 2011). This set of applications can then further be classified into *participatory sensing* (Burke et al., 2006) and *opportunistic sensing* (Lane et al., 2010) applications. Participatory sensing requires an active and conscious involvement of the user in order to contribute sensor data, while in opportunistic sensing, user involvement is minimal and sensor measurements as well as data transmission are done passively. In reality, mobile crowdsensing applications will often be located somewhere between these two extremes and use both paradigms to some extent. Furthermore, there exist recent works that reflect the categories of participatory and opportunistic sensing in the healthcare context (e.g., Pryss, 2019).

Furthermore, we consider the concept of mobile crowdsensing in the healthcare domain. Therefore, we are focusing on correlating personal sensing data with assessment data in order to gain insights on specific health conditions, (chronic) diseases and the patients' behavior. We consider the potential knowledge generated from this data as the phenomenon of common interest in terms of mobile crowdsensing. There are a number of applications in the field of healthcare (Guo et al., 2015). Its use cases include data collection in clinical and health/psychological trials (Pryss et al., 2015; Schobel et al., 2015), environmental monitoring and pollution measurement like noise pollution (Schweizer et al., 2011; Zappatore et al., 2017) or air pollution (Mun et al., 2009), public health (Wesolowski et al., 2012), and personal well-being (Consolvo et al., 2006). Although various mobile applications and solutions have been proposed, less works exist that cover reference settings to build generic solutions (Tokosi and Scholtz, 2019). In addition, few works are based on comprehensive experiences that are gained through various long-running projects (Tokosi and Scholtz, 2019).

### 2.2. Ecological Momentary Assessments (EMA)

Ecological Momentary Assessment (EMA) (Stone and Shiffman, 1994) denotes a range of research methods aiming to assess phenomena with ecological validity by allowing subjects and patients to repeatedly report in real time, in real-world settings, over time, and across contexts and therefore avoiding the bias of retrospective reports (Pryss et al., 2018a). Among numerous other aspects, EMA is characterized by several key features (Shiffman et al., 2008):

- Ecological: Data is collected *in situ*, i.e., in real-world settings and environments, which constitutes the ecological validity.
- Momentary: Assessments focus on current or very recent states in real time, which aims to avoid a bias associated with retrospective assessments.
- Strategic sampling: Assessment timings are strategically selected by specific sampling schemes, e.g., based on particular events of interest or by random, representative samplings across contexts.
- Longitudinal data: Subjects complete multiple assessments over time, which provides longitudinal data with insights on how the state varies over time and across situations.

A related methodology in the field of momentary research is the *Experience Sampling Method* (ESM) (Larson and Csikszentmihalyi, 2014; Van Haren, 2018), which aims at measuring momentary behavior, thoughts, symptoms, and feelings of participants, collected through self-reports that are typically filled out several times a day over several consecutive days (Myin-Germeys et al., 2009; Van Berkel et al., 2018). Generally, ESM has a focus on random time sampling and private, subjective experiences, while EMA is defined more broadly, as it also includes other sampling approaches and behavioral as well as physiological measures (Stone and Shiffman, 2002). Since we are striving to make our architecture as generic as possible and to additionally address physiological sampling via mobile sensors, we focus on EMA within the scope of this work.

#### 2.2.1. Implementation of EMA With Mobile Devices

EMA studies can be carried out with the help of portable electronic devices, which support the following EMA key functions (Shiffman, 2007; Shiffman et al., 2008):

1. Present assessment content to the subject (i.e., display questions and response options).
2. Manage assessment logic (e.g., handle branching and validate inputs).
3. Provide time-stamp data to document when assessments are completed.
4. Store assessment data.
5. Manage prompting schedules (e.g., determine when assessments should be made).
6. Prompt the subject to complete assessments.

Modern smartphones offer all of these functions, as they provide high-resolution displays, advanced processing power and storage, as well as push notifications (Raento et al., 2009). They have already been used in different EMA studies (Ebner-Priemer and Kubiak, 2007; Schlee et al., 2016). We summarize smartphone applications that offer EMA functionality using the term *EMA apps*. Smartphones offer additional capabilities that go beyond the initially defined EMA key functions, most importantly advanced processing capabilities, an (almost) always available network connection and built-in as well locally connected sensors (Van Berkel et al., 2018). Furthermore, data can be stored locally on the device and synchronized with the server, enabling an offline availability. Therefore, we explore different extensions of EMA apps and their combinations and study their effects. These extensions can be broadly categorized in (1) *guidance*, (2) *feedback*, (3) *adjustable prompts*, and (4) *dynamic questionnaires*. Generally, we distinguish between EMA apps and features that are used for data collection only (mainly research) and others that offer a benefit to the user (research and health care). The four categories of extensions we consider are described in the following:

- Guidance: We refer to *guidance* as the option for the user of the EMA app to link to a contact person. This contact person might be some kind of healthcare provider (HCP) that has some professional qualifications, e.g., a physician or therapist. The HCP might influence the process of EMA prompts, provide feedback to submitted data, and offer general advice to the user, or just act as an observer.
- Feedback: The EMA app could offer feedback to the user when he/she submits questionnaires. This feedback can be in the form of text messages by the HCP or automated feedback by the app, like tips and warnings when certain thresholds are exceeded, as well as graphical feedback in the form of graphs about the history of different measurements. We assume that feedback of this kind might act as an incentive to users and therefore increase adherence, but we also want to study the effects of this feedback on the EMA data.
- Adjustable prompts: Assessment prompts (i.e., notifications) can either be fixed and determined by the system, defined by the HCP, event-triggered (e.g., when a patient perceives his tinnitus, or when a context change is detected through sensor data), or can be adjusted by the user in a flexible manner.
- Dynamic questionnaires: The content of EMA questionnaires could be dynamic and adjusted depending on answered questionnaires in the past, occurring events, or other external parameters (e.g., the current weather retrieved through a web service).

#### 2.2.2. Potential Challenges

There are a number of potential challenges when employing EMA studies, which are outlined in the following (Van Berkel et al., 2018):

- Participant burden: Answering questionnaires multiple times a day can be burdensome for participants. To counteract this issue, the number of questions, alerts, and question types should be kept as small as possible.
- Participant retention: Related to the frequent answering of questionnaires, study dropout rates are generally high. There has to be some sort of incentive for participants in order to keep them entering their data in a constant manner.
- Programming: There is no generic software solution that allows to employ EMA studies on mobile devices without requiring at least basic programming skills.
- Platform heterogeneity: Flexible software is required in order to support a large number of different hardware devices and operating systems.
- Data quality: Since data is not collected in a controlled environment, participants' data might be of low quality or noisy. Mechanisms should be in place to avoid or compensate missing, wrong or careless answers, as well as response shifts (i.e., changes in the participant's internal standards) or changes in the participants reactivity (i.e., behavioral adjustments because the participants know that they are being observed). In the context of participant retention, participants might answer the questionnaires as often as possible, even in a dishonest way, if they expect a reward or think they are supporting the platform in this way.

### 2.3. Combining Mobile Crowdsensing and Ecological Momentary Assessments

**Figure 1:**
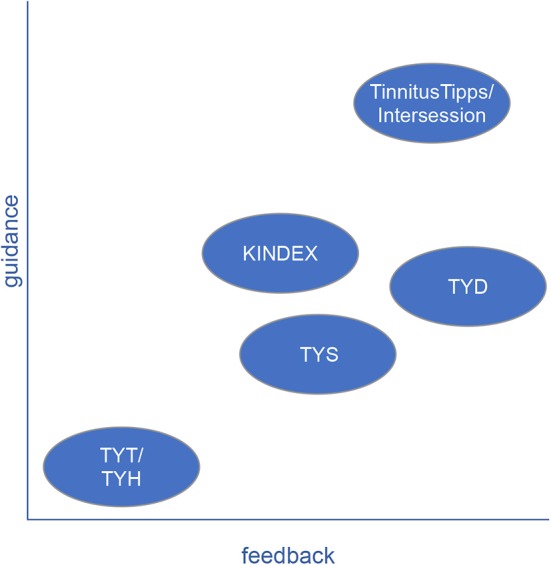
Subjective and relative rating of guidance and feedback of selected EMA apps developed by the authors.

Crucially, smartphones enable us to not only collect explicit answers to EMA questionnaires, but additionally capture the *context* in which they are collected (Van Berkel et al., 2018). We consider EMA apps similar to mobile crowdsensing, in which the assessed phenomenon in terms of mobile crowdsensing is the ecological data collected in EMA questionnaires. Consequently, we combine the concepts and features of EMA apps with the paradigms of mobile crowdsensing by correlating questionnaire responses and sensor data in order to gain new insights on certain phenomena. Furthermore, we derive different classes of mobile crowdsensing EMA apps depending on the EMA features they provide and the crowdsensing paradigms that they make use of. Table 1 shows examples for apps that are incorporating both EMA and mobile crowdsensing features that were developed by the authors. The *TrackYourTinnitus (TYT)* project tracks one's individual tinnitus and is described in detail in section 3. Similar to TYT, *TrackYourHearing* (TYH), *TrackYourDiabetes (TYD)*, and *TrackYourStress (TYS)* (Pryss et al., 2019) help the user to assess and track the progress of their hearing loss, diabetes, or stress level, respectively, and allow them to be more sensitive to symptom changes in specific contexts. The *TinnitusTipps* app was designed to enable the communication between healthcare providers (HCP) and tinnitus patients, including the assessment of the user's tinnitus and various automatic as well as manual feedback options. The *KINDEX mum screen* enables the assessment of psychosocial stress factors during pregnancy (Ruf-Leuschner et al., 2016). Finally, the *Intersession* app focuses on the assessment and guidance of users during the time between therapy sessions. Even though the last two apps are not incorporating any sensor measurements and are therefore by definition not utilizing MCS, we consider their assessed ecological data as phenomenon of common interest in terms of mobile crowdsensing and their contributions regarding guidance and feedback as a valuable basis for MCS-EMA platforms. The number of users, submitted answer sheets and released versions as well as the incorporated sensor measurements for the developed apps are shown in Table 2. The sensor measurements are performed while the patients answer the questionnaires and stored together with the answer data in order to allow to investigate correlations. To put these apps into perspective, Figure 1 shows how they are incorporating guidance and feedback (as defined in section 2.2.1) on a relative two-dimensional scale based on a subjective rating (however, guided by the extensive experiences) by the authors.

**Table 1:** Examples of apps developed by the authors combining mobile crowdsensing (MCS) and ecological momentary assessments (EMA), compared according to their respective features.

**App name**	**Guidance**	**Feedback**	**Adjustable prompts**	**Dynamic questionnaires**	**Participatory sensing**	**Opportunistic sensing**
TrackYourTinnitus (TYT)			✓	✓	✓	
TrackYourHearing (TYH)^a^			✓	✓	✓	
TrackYourDiabetes (TYD)	✓	✓	✓		✓	
TrackYourStress (TYS)^b^	✓	✓	✓		✓	
TinnitusTipps	✓	✓	✓	✓	✓	
KINDEX	✓	✓		✓	(✓)	
Intersession	✓	✓	✓	✓	(✓)	

a https://www.trackyourhearing.org/

b https://www.trackyourstress.org/

**Table 2:** Descriptive statistics on mobile crowdsensing EMA apps developed by the authors.

**App**	**Number of total users**	**Number of users with at least one answer sheet^†^**	**Submitted answer sheets**	**Sensor measurements**
TrackYourTinnitus (TYT)	4,480	2,905	76,105	Environmental sound level
TrackYourHearing (TYH)	437	167	6,102	Environmental sound level, EEG^*^
TrackYourDiabetes (TYD)	58	36	3,097	Position (GPS), environmental sound level, blood sugar^*^
TrackYourStress (TYS)	204	138	2,989	Position (GPS), environmental sound level, heart rate sensor
TinnitusTipps	95	66	8,209	Position (GPS)
KINDEX	1,779	1,779	1,943	–
Intersession	6	4	220	–
Total	7,059	5,095	98,665	

*Numbers extracted on 05 Dec 2019*.

* *External sensor measurements*.

† *Compared to the second column, this column does not include users that quit using the app after registration and are therefore considered as early dropouts*.

## 3. Lessons Learned From the TrackYourTinnitus Project

The TrackYourTinnitus (TYT) platform is available and has been maintained since April 2014. It consists of a website for registration^1^, two native mobile applications (iOS and Android), and a central backend that stores the collected data in a relational database. The mobile apps track the individual tinnitus perception by asking the patients to complete tinnitus assessment EMA questionnaires at different times during the day and on a random basis. The daily questionnaire is assessing tinnitus by measuring eight dimensions, e.g., tinnitus loudness and distress, utilizing the questions shown in Table 3. Furthermore, the apps measure the environmental sound level while patients fill out the questionnaires (Pryss et al., 2015). Medically, tinnitus is the perception of a sound when no corresponding external sound is present. The symptoms, in turn, are subjective and vary over time. Hence, TYT was realized to monitor and evaluate the variability of symptoms over time based on EMA and mobile crowdsensing (Schlee et al., 2016).

**Table 3:** Questions of the daily questionnaire in the TrackYourTinnitus (TYT) smartphone application, along with their scale and the dimension they measure (Schlee et al., 2016; Pryss et al., 2017).

**#**	**Question**	**Scale**	**Dimension**
1	Did you perceive the tinnitus right now?	BS	Perception
2	How loud is the tinnitus right now?	VAS	Loudness
3	How stressful is the tinnitus right now?	VAS	Distress
4	How is your mood right now?	VAS	Mood
5	How is your arousal right now?	VAS	Arousal
6	Do you feel stressed right now?	VAS	Stress
7	How much did you concentrate on the things you are doing right now?	VAS	Concentration
8	Do you feel irritable right now?	BS	Irritability

*BS, binary scale; VAS, visual analog scale*.

One potential risk worth considering is whether continuous tracking of tinnitus with the app could aggravate the patient's symptoms by drawing additional attention to them. However, it has been shown that the regular use of the TYT app has no significant negative effect on the perceived tinnitus loudness and the tinnitus distress. Therefore, the app can be considered as a safe method for the longitudinal assessment of tinnitus symptoms in the everyday life of patients (Schlee et al., 2016). Another health risk is that patients (or their HCP) use TYT as a treatment tool and unnecessarily change their treatment plan due to self-reported symptoms in the app. In order to make patients aware of these risks, they are outlined on the TYT website^2^.

**Figure 2:**
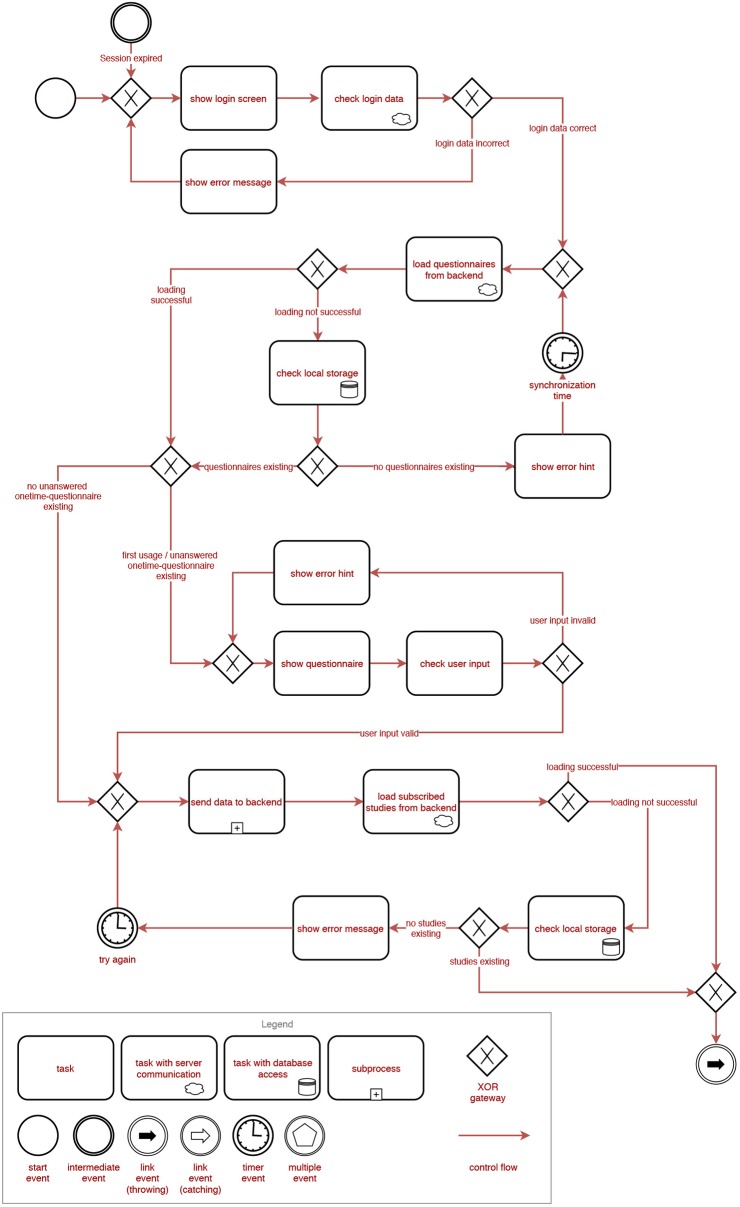
BPMN representation of the general process of the TrackYourTinnitus (TYT) smartphone application (Part 1).

**Figure 3:**
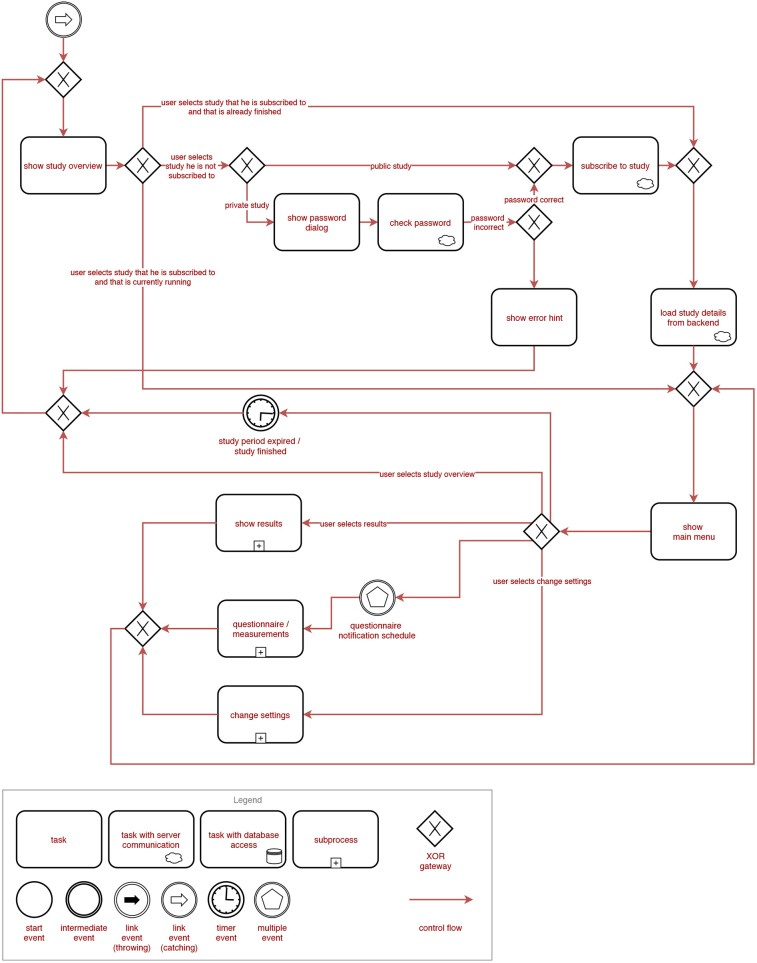
BPMN representation of the general process of the TrackYourTinnitus (TYT) smartphone application (Part 2).

Figures 2, 3 show the general process a user is going through when using the TYT iOS or Android application. Note that these figures are process-oriented graphs in terms of the Business Process Modeling Notation (BPMN). This notation is an industry standard and also well-known for the documentation of healthcare-related procedures (Reichert and Pryss, 2017). With respect to these figures, first of all, a user authenticates himself/herself with his/her login data. Then, all available questionnaires are loaded from a central backend. If the loading is unsuccessful (e.g., no connection to the server can be established), locally stored data is used until the next synchronization attempt. In case there are no locally stored questionnaires, the synchronization attempt is retried until it succeeds. The app then checks if there are *first usage* (i.e., questionnaires that are only answered once after the first login) or *one-time* (i.e., questionnaires that are only answered once but might be answered at a later time) questionnaires available. If this is the case, these questionnaires are displayed and can be filled in by the user one after the other. Data is then synchronized with the backend by uploading all newly answered questionnaire data and loading all studies the user is subscribed to. If the synchronization is unsuccessful, the local storage is checked once again and the process is retried after some time if no data can be retrieved both remotely and locally. In the next step, an overview of all available studies is presented to the user. He/She may then select a study from that overview. Depending on the study and the user's subscription status, the following process differs. If the user is currently not subscribed to the study, he/she will be able to (a) directly subscribe to that study if it is *public*, or (b) be prompted to enter a password if it is a *private* study. For private studies, the password is then checked with the backend. If the password is correct, the user is subscribed to the study. Otherwise, an error hint is displayed and the user is redirected back to the study overview. If the user is currently subscribed to the study and that study is already finished, its details are loaded from the backend and the user is forwarded to the main menu. If the user is currently subscribed to the study and that study is still running, the user is also forwarded to the main menu. From the main menu, the user can choose to go back to the study overview, display his/her results, fill in questionnaires and perform sensor measurements, and finally, change the settings. From the results, questionnaire and settings views, he/she can always return to the main menu. If the user selects the study overview, or if the study period is expired (respectively, if the study is finished), the study overview is displayed once again.

During the development and advancement of the platform, we faced several challenges and peculiarities. Additionally, we gained some valuable insights when implementing such a combination of an EMA and MCS approach. First, we required a basic functionality to identify different users. One could argue that, since data has to be stored anonymized, a device ID would be sufficient, but this would prevent the users from changing devices without data loss. Therefore, we implemented basic authentication and authorization mechanisms, including registration via email, login with username and password, as well as password reset features.

The core of the application is the presentation and fill-in process of (EMA) questionnaires. In order to facilitate adding new questionnaires and adjusting existing questionnaires at a later time, the platform should offer a generic approach to handle questionnaires. We achieved this by defining the questionnaires as *JavaScript Object Notation (JSON)* objects containing an array of *questionnaire elements* (e.g., headline, text, multiple-choice-question), stored on the backend. The apps provide components with functionalities to render, configure, and handle the input for each of these elements. The components are then put together in a *list view*, and additional checks like input validation or ensuring that required questions are filled in are performed. Another requirement was to make the questionnaires easy to use, while not introducing bias. In order to improve usability of the apps, we tried to make the questionnaires look similar to their paper-pencil counterpart while using as many system-provided and default UI elements as possible when implementing the element components. However, some of the default UI elements are not suitable for the use in psychological questionnaires and had to be adjusted. For instance, default iOS and Android sliders have a pre-selected value, which fosters undesirable anchoring affects (Tversky and Kahneman, 1974).

Furthermore, a sophisticated algorithm has to be deployed in order to implement the notification (i.e., prompting) schedules. The algorithm has to account for the users' sleep and work schedules, ensuring notifications are not too close to each other^3^ and allowing different adjustments by the user (e.g., the time frame and number of notifications per day). Since we managed the notification schedules exclusively inside the apps, there was no way to retrieve any information on the scheduled and received notifications. Therefore, we were unable to extract valuable information on how users change their notification schedules and, most importantly, we could not evaluate the notification adherence. We offered both *random* (in a given time frame, adjustable by the user) and *fixed* (at an exact point in time, chosen by the user) notifications. However, users reported problems with random notifications not being delivered as configured or not delivered at all. While fixed notifications have proven to be more reliable, more flexible, and less disruptive to the user, their value in terms of EMA is to be questioned. Users might integrate answering the questionnaire into their daily routine, which can lead to a possible bias.

In our first version of the app, we incorporated an environmental sound measurement. If enabled by the user, the app tracks the average loudness recorded by the smartphone microphone while the user answers the questionnaires. This value is then stored together with the questionnaire data and can be correlated to gain new valuable insights on tinnitus and its interrelations with environmental sound. However, due to manufacturer and device model differences, measurements are not comparable across users. Calibrations with different device models or other measures to ensure comparability should be performed before integrating similar measurements into mobile applications. Additionally, these sensor measurements are hard-coded into the apps. A dynamic framework to integrate internal and external sensors would facilitate studies aiming to correlate different sensor data with questionnaire data. In this way, one could integrate additional sensors, e.g., positioning with GPS in order to investigate the interrelations to motion patterns or the influence of weather-related factors.

Another aspect worth considering is *incentives*. There needs to be some sort of motivation for users to continuously submit data. Zhang et al. (2015) divide incentives in mobile crowd sensing applications into entertainment, service, and monetary incentives. Since we do not consider monetary incentives sustainable in the long term (especially in the research context), we focus on the former two categories in order to increase the users' extrinsic and intrinsic motivation. While in TYT, we provided some minimalistic feedback in the form of a chart of the perceived tinnitus loudness and an option to review the history of submitted questionnaires for each individual user, we believe the main incentive for users is the contribution to research on a chronic disorder from which they are suffering. However, more than 78% of users drop out after 10 days of participation. More incentive mechanisms, like advanced feedback, gamification, or social features should be implemented (Agrawal et al., 2018).

In order to perform different studies with the app (and to exclude test users from the actual data set), the need to separate users into study groups inside the app emerged. We updated the app to incorporate a basic study allocation. Users are able to join studies by manually selecting them from a list inside the app. However, users can currently only be member of a single study at a time and there is no functionality in place for the study manager to control or verify which user joins which study without checking the database.

Since mobile devices are not guaranteed to always be connected to the internet (i.e., *be online*), the app should also be functional without internet connection whenever possible. TYT offers a basic *offline functionality* by initially downloading all questionnaires and storing them on the device. Additionally, the users' given answers for questionnaires are cached on the device if there is no internet connectivity until the connection is restored. This way, the feedback features also remain functional. However, other features, e.g., the study management, are only available if the device is online.

Furthermore, *safety, security, and privacy* are aspects of high importance in the healthcare domain. Region-specific regulations, e.g., the *General Data Protection Regulation (GDPR)* and the *Medical Device Regulation (MDR)* in the EU, as well as high expectations of patients need to be considered when designing a software system in this field. TYT applies state-of-the-art security measures with an email verification as part of the registration process, credential-based authentication and token-based authorization (see above) as well as encrypted data transmission via SSL/TLS (Rescorla, 2018). Health risks are outlined on the website. However, since safety, security, and privacy requirements are constantly evolving, a more transparent informed consent, additional security measures and a privacy-preserving design would be desirable for the future (e.g., Beierle et al., 2019).

*Data quality* of the submitted data is another critical issue in MCS-EMA apps (see section 2.2.2). As already discussed above, reliable sensor and comparable measurements on mobile devices are difficult to achieve due to the variety of device models. But, also for the questionnaire data, no real statement can be made regarding its quality. Since we only require the user to answer two of the eight questions in the daily questionnaire, users can skip most of the questions if they would like to do so, which leads to missing values. Also, if users feel forced to answer a question or have malicious intentions, they might provide untruthful data. In addition, the use of a smartphone to gather large amounts of personal data in real life that is stored to a large database for scientific research could boost competition thoughts. Consequently, participants might provide data only for the purpose of providing more data than others. Such factors should be taken into account and mechanisms should be in place to cope with data quality.

Moreover, scientists providing the platform and HCPs want to analyze the collected data. In TYT, data analysis is only possible in a static way by querying the raw database. More flexible, on-demand analysis functionalities for scientists evaluating the platform data are desirable. Furthermore, HCPs and their patients could benefit from a dynamic analysis of the patients' data, providing detailed insights and building the baseline for tailored feedback.

**Figure 4:**
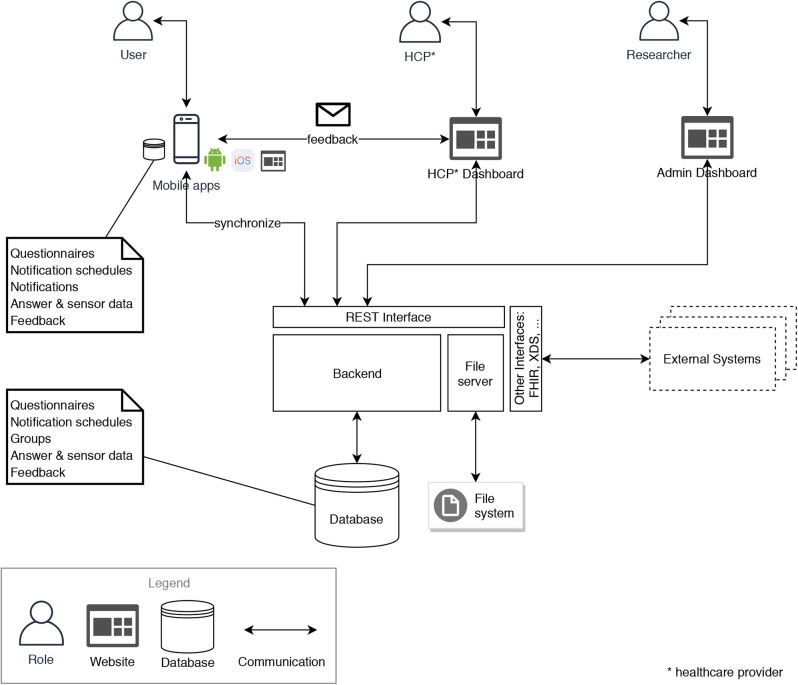
Reference architecture for MCS-EMA platforms in the healthcare domain.

Finally, the experiences gained with TrackYourTinnitus and the projects shown in Table 2 are discussed in the light of their general contribution and their generalizability. A recent review of mobile health crowdsensing research (Tokosi and Scholtz, 2019) shows that the projects shown in Table 2 and the related papers are heavily recognized by their selected key terms of existing works. Tokosi and Scholtz (2019) also shows that although more and more research is pursued in this context, less experiences are reported that were gained over multiple large-scale and long-running projects. Therefore, we consider our experiences as a proper starting point to conceive a reference architecture that incorporates aspects that are relevant on one hand. On the other, these aspects have shown their importance at multiple times. Furthermore, the authors have already worked on better generic solutions for parts of the reference architecture. For example, for the REST interface (see Figure 4) in Pryss et al. (2018b), a more generic solution was proposed. This solution, in turn, is utilized by all projects shown in Table 2 that have been started after TrackYourTinnitus. However, as for other purposes, like mobile data collection, better generic solutions have been proposed (e.g., Schobel et al., 2019). A configurable crowdsensing platform based on (1) the archetype shown in (Schobel et al., 2019) and (2) the results of this work is currently conceived. Moreover, developments, such as *PACO*^4^ show that easily customizable MCS-EMA apps are highly welcome by users. In addition, commercial tools, such as *ilumivu*^5^ emphasize the need of generic solutions in the given context of EMA and mobile crowdsensing. Thereby, the ilumivu technical solution provides already sophisticated features for EMA apps on a generic level. Importantly, these features deal with many aspects raised in this work. On the other, ilumivu still does not consider all of the discussed aspects. For example, ilumivu does not convey how they cope with a management of incentives. Following this, the work at hand can be utilized to reflect existing solutions or new developments with the shown experiences and derived recommendations, especially as they are gained over time and across projects. We do not claim that these recommendations are complete or cover every aspect, but we consider them as a proper starting point for various projects and questions in the context of healthcare and the combination of mobile crowdsensing and EMA.

## 4. Toward a Reference Architecture

Based on the findings in section 3, we derive a number of recommendations for a mature and contemporary MCS-EMA platform. We then propose a reference architecture to address these recommendations and discuss technical considerations with respect to the implementation.

### 4.1. Recommendations

We derived twelve recommendations from the lessons learned during the TYT project (see section 3), various discussions with colleagues and domain experts, as well as general considerations when building a modern software system. Namely, these recommendations are (R1) *User Identity*, (R2) *Generic Questionnaires*, (R4) *Sensors and Context-Awareness*, (R5) *Incentive Mechanisms*, (R6) *Groups, Studies and HCPs*, (R7) *High Availability and Performance*, (R8) *Offline Availability*, (R9) *Safety, Security, and Privacy*, (R10) *Data Quality*, (R11) *Data Analysis*, and (R12) *Interoperability*. The recommendations are described in detail in Tables 4, 5.

**Table 4:** Recommendations for a platform combining mobile crowdsensing (MCS) and ecological momentary assessments (EMA) in the healthcare domain (Part 1).

**ID**	**Name**	**Description**
R1	User identity	The platform should allow authentication and authorization in order to uniquely identify users. The user should be able to log into the platform with multiple devices, change and recover his/her password if it is lost, and deactivate as well as delete his/her account.
R2	Generic questionnaires	The platform should be able to handle generically defined questionnaires. Both one-time (e.g., demographic) and repeating (e.g., EMA) questionnaires should be supported. The mobile application should be able to display multiple questionnaires, which are available at different intervals, concurrently. Supported question types should be at least *single choice, multiple choice, text input*, and *date input*. There should be an option to define dynamic questionnaires, which adapt to the previous input of the user (i.e., *conditional content*). Optionally, the user can also adapt his/her own questionnaire according to his/her needs (e.g., add additional questions).
R3	Notifications	The platform should be able to prompt the user to fill in questionnaires. For each questionnaire, one or multiple *notification schedules* can be defined, which determines how and how often the user is notified. A default configuration for each questionnaire can be provided, which is optionally adjustable by the user. Notifications can be set for fixed times (i.e., *fixed*), or randomly within a given time frame for each day (i.e., *random*). An algorithm should ensure that notifications from different schedules are not conflicting with each other. Additionally, notifications that are event-triggered (e.g., by a context change) can be defined. Information on the notification adherence (i.e., when the notification has been displayed; if/when did the user trigger the notification) should be stored and made available for analysis.
R4	Sensors and context-awareness	For each questionnaire, a set of sensor measurements (e.g., GPS coordinates, sound level, brightness, or wearable sensors) that are performed on the mobile devices should be definable. These measurements can be configured to be performed (a) once or (b) continuously during the fill-in process of the respective questionnaire; (c) continuously during the app usage; or (d) continuously in the background. Additionally, different sensors can be combined (i.e., *sensor fusion*) to retrieve various context information.
R5	Incentive mechanisms	Different incentive mechanisms should be deployed in order to support the patients' adherence. We define three types of incentives: *feedback, gamification*, or *social features*.
R5.1	Feedback	The platform should provide different types of feedback to the user. Graphical feedback (e.g., charts or graphs), daily tips, automatic feedback based on the given answers, as well as manual feedback in the form of messages by the HCP can be incorporated. Manual feedback could be supported or partly be replaced by incorporating a chatbot with automated analysis of the user's input (both answer data and text messages).
R5.2	Gamification	The platform should offer gamification features like achievements (e.g., submission streaks), badges, points, and leaderboards.
R5.3	Social features	The platform should offer social features like public user profiles, group chats, discussion boards on certain topics and following as well as sharing functionalities.

**Table 5:** Recommendations for a platform combining mobile crowdsensing (MCS) and ecological momentary assessments (EMA) in the healthcare domain (Part 2).

**ID**	**Name**	**Description**
R6	Groups, studies, and HCPs	Users should be able to join one or multiple groups. These groups can represent studies, HCPs or other groupings (e.g., test users). Users can be invited to groups by their respective group owner (e.g., the HCP) or join them via different join mechanisms (e.g., join requests, password-restricted or freely).
R7	High availability and Performance	The platform should be available to its users in the best possible way. There should not be any noticeable performance drops under higher loads.
R8	Offline availability	The mobile app should still be functional when there is no internet connection (or more generally, no connection to the server) whenever possible. All data should be stored on the device where appropriate and synchronized with the server.
R9	Safety, security, and privacy	The platform should meet high safety, security and privacy standards. Region-specific regulations like the *EU General Data Protection Regulation (GDPR)* and the *Medical Device Regulation (MDR)* should be considered. All confidential data should be stored securely and transmitted in encrypted form. User data and credentials should be stored separately from the answer data. Health risks should be identified and addressed at an early stage and outlined to users and HCPs in a transparent way. A security model for the mobile apps and the entire platform should exist.
R10	Data quality	Data quality should be kept as high as possible. Different data quality aspects like believability, relevancy, accuracy (i.e., error-free, reliable, precise), interpretability, understandability, accessibility, objectivity, timeliness, completeness and (representational) consistency (Wang and Strong, 1996) should be addressed depending on the specific requirements of the use case. The platform should perform input validation and prevent invalid inputs, perform plausibility checks, as well as other measures to improve quality of answer and sensor data. This also includes measures for detecting and handling misstatements by users, which might be both intentional and malicious (e.g., *faking*), as well as unintentional (e.g., *self-deception*), summarized with the terms *faking* and *socially desirable responding (SDR)* (Paulhus, 2001; Van de Mortel, 2008).
R11	Data analysis	The platform should offer easy-to-use data analysis functionalities on live data for researchers, HCPs, and also the users themselves. Both static and dynamic data analysis (e.g., aggregation with the help of filters and time windows or clustering) should be enabled. All relevant data should be exportable to common formats (e.g., CSV, SPSS, R, PDF). The HCP and the user should be able to review and analyze the individual answers to questionnaires as well as sensor measurements and compare them to the data of other users.
R12	Interoperability	The platform should offer a good interoperability with other (external) systems. This includes implementing common data exchange format standards and communication protocols, as well as providing uniform, understandable, and well-documented interfaces.

### 4.2. Architecture

Based on the recommendations defined in section 4.1, we propose a reference architecture for a platform supporting the combination of mobile crowdsensing and ecological momentary assessments in the healthcare domain. Figure 4 shows the general architecture. It comprises a central backend with different services, a database and a file server, as well as mobile apps for both Android and iOS, a web dashboard for HCPs and another web dashboard for system administrators (*admins*). The clients (mobile apps, HCP dashboard and admin dashboard) communicate with the backend via a RESTful interface. Files, like multimedia and documents, are stored on a file server. Relevant files are downloaded and additionally stored on the mobile devices. All relevant data, like questionnaires, notification schedules as well as answer and sensor data are synchronized between the central database in the backend and the mobile apps' local databases. The backend additionally provides other interfaces for external systems, implementing common standards in the healthcare domain.

### 4.3. Selected Technical Considerations

**Figure 5:**
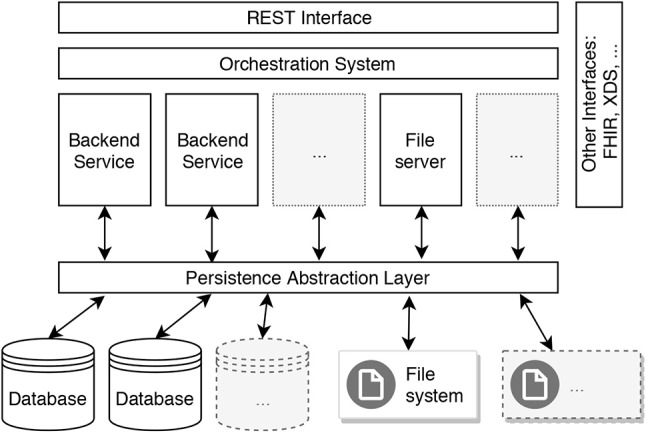
Scalable design of a backend in the reference architecture for MCS-EMA platforms in the healthcare domain.

Furthermore, we discuss technical considerations in order to address some of the architectural aspects of the defined recommendations in respect to our reference architecture. First, in order to achieve high availability, the system has to be scalable, and in the best case, elastic. According to definitions provided by Herbst et al., *scalability* is “the ability of a system to handle increasing workloads with adequate performance,” while *elasticity* is “the degree to which a system is able to adapt to workload changes by provisioning and deprovisioning resources in an autonomic manner, such that at each point in time the available resources match the current demand as closely as possible” (Herbst et al., 2013). We suggest to use a *cloud-native* approach to address these recommendations. A *cloud-native application (CNA)* is explicitly designed to be operated in the cloud. Therefore, such application is—by design—distributed, elastic, and horizontally scalable. Furthermore, it is composed of microservices with a minimum of isolated states (Kratzke and Quint, 2017). The internal architecture for a cloud-native implementation of the backend in our reference architecture is shown in Figure 5. The backend can be decomposed to multiple microservices, and these microservices can then be replicated in order to enable horizontal scalability. Optimally, the database, file server and file system should be distributed and/or replicated as well. In order to provide elasticity, an *orchestration system* is used to monitor metrics describing the load of the system and automatically orchestrate resources based on these metrics in order to scale in and scale out. A common approach would be to use Docker^6^ as container technology to implement microservices and Kubernetes^7^ (Burns et al., 2016) as container-orchestration system.

In order to provide high levels of security and privacy, all communication between different components of the architecture should be encrypted. All personal and private user data should be stored separately from the application data to reduce the risk of it being exposed in case of a data breach. Optionally, in a *privacy-preserving design*, this data should be encrypted in a way that it can only be decrypted by each respective user himself. In the best case, a dedicated privacy model is incorporated or developed (e.g., Beierle et al., 2019).

Furthermore, for the development of the mobile apps, it has to be decided whether to develop a native app for each target platform (e.g., Android, iOS, web browser) or use cross-platform frameworks that enable the developer to use a single code-base and deploy this code to different platforms. We recommend to use cross-platform frameworks (e.g., *Xamarin*^8^, *Flutter*^9^, or *Ionic*^10^) for small developer teams and teams which are prone to changes (e.g., research projects), since the single code base requires less efforts for development and maintenance, as well as causes lesser heterogeneity-based challenges in programming languages and tools, which makes it easier for new developers to enter the team. However, for bigger and more consistent developer teams, native app development might be better suited. Native apps might provide a better interface to the operating system and therefore more control over sensors and the user interface, as well as potentially better performance. This has special value to MCS apps incorporating advanced sensor usage.

Finally, in order to provide good interoperability with other internal as well as external systems, common interfaces should be provided. This includes state-of-the-art architectural styles in web technology like *REST* (Fielding and Taylor, 2000; Pryss et al., 2018b), but also standards in the healthcare domain [e.g., *FHIR*^11^ or *XDS* (Trotter and Uhlman, 2011)]. Standards that one wants to support should be considered at an early stage when designing the data models.

## 5. Discussion

We argue that, when considering mobile crowdsensing in the healthcare domain, differentiating only between participatory and opportunistic sensing is not sufficient. Other aspects like context-awareness, incentive mechanisms, groups, security, and privacy, data quality, as well as technical aspects like availability, performance, offline availability and interoperability should be also thoroughly taken into account. Additionally, although personal sensing data on its own only belongs to an individual user, it can be used in order to be beneficial for the community as a whole by processing, clustering, and correlating this type of data. Therefore, we further argue that in the context of mobile crowdsensing in healthcare, there is no distinct separation between community sensing and personal sensing, and that both concepts should be considered depending on the scenario that is addressed.

Furthermore, in the literature, MCS and EMA are considered as separate, mostly unrelated concepts. While they have different origins, we argue that both concepts make use of similar approaches, namely leveraging the crowd and their (already existing) mobile devices in order to assess phenomena of common interest. Therefore, they should be considered closely related to each other, and their combination should get more awareness. Beyond that, the architectural model is often not provided in publications on MCS and EMA studies, although we argue that it has meaningful implications on the comparability of their results. We believe that a reference architecture, such as that introduced in this work, can raise awareness and counteract this issue to a certain degree. In this context, we have particularly shown which aspects the reference architecture incorporates to develop more generic technical solutions based on it.

## 6. Conclusion

In this work, we discussed the combination of mobile crowdsensing (MCS) and ecological momentary assessment (EMA) in the healthcare domain. We introduced both terms and described how we considered their underlying concepts that are similar to each other, which fosters combining MCS and EMA in a single approach. Furthermore, we discussed the lessons we learned from the TrackYourTinnitus project, which is running for over 5 years. Based on these findings, we derived recommendations for a platform supporting the combination of MCS and EMA in the healthcare domain. We then proposed a reference architecture for such a platform, described its components and how they interact. Additionally, we outlined how the reference architecture could be implemented in order to address the defined recommendations from the technical side. Furthermore, we discussed how MCS and EMA research should be considering both concepts in combination and propose that publications in this field should refer to the used architectural model.

In conclusion, one can see that there are numerous conceptual, architectural and technical, as well as legal challenges when designing a MCS-EMA platform for the healthcare domain. We believe that the defined recommendations can—adjusted to the individual factors, needs and requirements of a (research) project or product—act as foundation for future MCS-EMA systems. All the different aspects should be considered at an early stage of the project. Additionally, the reference architecture can serve as a generic template for a platform implementation. Technical considerations should be kept in mind in order to be able to scale and cope with future requirements. However, we believe that the combination of MCS and EMA is a promising approach for many different use cases in the healthcare domain. For this endeavor, our reference architecture and recommendations shall be a basis for more generic and comparable technical solutions.

## Author Contributions

RK and RP substantially contributed to the TrackYourTinnitus platform, drafted, and revised the manuscript. WS, MS, MR, BL, and TP substantially contributed to the TrackYourTinnitus platform and revised the manuscript. HB read and revised the manuscript. RH substantially contributed to the TinnitusTipps platform and revised the manuscript.

### Conflict of Interest

RH was employed by the company Sivantos GmbH, Germany. The remaining authors declare that the research was conducted in the absence of any commercial or financial relationships that could be construed as a potential conflict of interest.

## Footnotes

^1^https://www.trackyourtinnitus.org/

^2^https://www.trackyourtinnitus.org/about

^3^We chose 15 min as minimal distance between two consecutive notifications.

^4^https://pacoapp.com/

^5^https://ilumivu.com/

^6^https://www.docker.com/

^7^https://kubernetes.io/

^8^https://dotnet.microsoft.com/apps/xamarin

^9^https://flutter.dev/

^10^https://ionicframework.com/

^11^https://www.hl7.org/fhir/
